# Exploring the relationship between eco-anxiety and suicide risk in adolescents with mental health disorders: insights from a cross-sectional observational study

**DOI:** 10.3389/fpsyg.2024.1408835

**Published:** 2025-01-07

**Authors:** Apolline Lerolle, Jean-Arthur Micoulaud-Franchi, Pierre Fourneret, Alexandre Heeren, Christophe Gauld

**Affiliations:** ^1^Service Psychopathologie du Développement de l'Enfant et de l'Adolescent, Hospices Civils de Lyon & Université de Lyon 1, Lyon, France; ^2^University Sleep Medicine Department, University Hospital of Bordeaux, Bordeaux, France; ^3^UMR CNRS 6033 SANPSY, University Hospital of Bordeaux, Bordeaux, France; ^4^Institut des Sciences Cognitives Marc Jeannerod, UMR 5229 CNRS & Université Claude Bernard Lyon 1, Lyon, France; ^5^Psychological Science Research Institute, UCLouvain, Louvain-la-Neuve, Belgium; ^6^Institute of Neuroscience, UCLouvain, Brussels, Belgium

**Keywords:** eco-anxiety, climate anxiety, suicide, adolescent, anxiety, depression

## Abstract

**Introduction:**

A limited number of studies have explored the connection between eco-anxiety, anxiety, and depression in adolescents. However, the relation between eco-anxiety and suicide remains unexamined. This cross-sectional observational study aims to bridge this gap by investigating the correlation between eco-anxiety intensity and suicide risk severity in adolescents.

**Methods:**

We used validated French versions of the Climate Anxiety Scale (CAS) and its two key dimensions (cognitive and emotional and functional impairments), alongside the Columbia Suicide Severity Rating Scale (C-SSRS) and the Hospital Anxiety and Depression scale (HAD).

**Results:**

Our study involved 87 hospitalized adolescent patients, aged 12–16. Although, the univariate model indicated a significant association between the CAS and the C-SSRS (β = 2.58; *p* = 0.049), the cognitive/emotional difficulties and functional impairment dimensions of eco-anxiety, considering different confounding factors, did not show statistical associations with the severity of suicide risk (respectively, *p* = 0.81 and *p* = 0.76).

**Discussion:**

In an expansive literature, these results show for the first time that eco-anxiety may not be the priority of adolescents seen by adolescent psychiatrists. Such an observation would imply not overmedicalizing a dimension of life which perhaps does not fall solely within the field of medicine, but which concerns environmental issues broader than medical field. However, an ethical and prudent approach in mental health care for this particularly fragile population remains necessary. This intersection of eco-anxiety and suicide in youth opens up new avenues of research in the realm of environmental and mental health studies.

## 1 Introduction

Suicide is a major public health problem. More than 6,000 suicide deaths were reported in 2017 among U.S. adolescents and young adults from 15 to 24 years of age (AHR, 2017). Suicide is the second cause of death among individuals 10–34 years of age [Centers for Disease Control and Prevention (CDC), 2018]. Thus, suicide among adolescents is a major public health priority due to its severity, frequency, and potentially preventable nature (Turecki et al., 2019).

Regarding risk factors associated with suicide risk, anxiety has been identified as one of the most important factors in adolescence (Bentley et al., 2016). In addition to being one of the most unanimously recognized risk factors of suicide (National Suicide Prevention Lifeline, 2015), anxiety is at the heart of a number of theories of suicide, based on cognitive models (Wenzel and Beck, 2008), interpersonal models (Joiner, 2007) or aversive self-awareness models (Baumeister, 1990). Recently, several national and international studies have indicated that climate change is a particularly stressful source of anxiety for adolescents and young adults (Hickman et al., 2021; Monsour et al., 2022; Ediz and Yanik, 2023; Vamvalis, 2023), a phenomenon referred to as eco-anxiety. Eco-anxiety is an umbrella term that refers to different definitions, e.g., a chronic fear of environmental catastrophe (Clayton et al., 2017), a mental distress or anxiety associated with worsening environmental conditions (Usher et al., 2019; Wullenkord et al., 2021), or a generalized feeling that the ecological foundations of existence are about to collapse (Albrecht, 2012). Whatever the meaning, it refers to a distress related to the fear of climate change and other environmental crises (such as biodiversity loss, pollution, or deforestation) (Watts et al., 2021), which can have harmful consequences by impairing daily life functioning (e.g., the ability to go to school or socialize) and scaring one's view of their future (Heeren and Asmundson, 2023). For example, in a recent large-scale study, approximately 75% of adolescents reported that they believed they had no future and that humanity is doomed (Hickman et al., 2021). Thus, it should come as no surprise that eco-anxiety has been associated with detrimental mental health outcomes, such as depression and general anxiety in adults (e.g., Clayton and Karazsia, 2020; Stanley et al., 2021; Mouguiama-Daouda et al., 2022). Nevertheless, uncertainty remains regarding these outcomes in adolescents.

Crucially, recent metric research about eco-anxiety has emphasized the importance of distinguishing between the potential adaptive and maladaptive features of eco-anxiety. For example, research on the “Climate Anxiety Scale” (CAS) (Clayton and Karazsia, 2020), one of the most widely used eco-anxiety assessment instruments in the world, included two distinct dimensions, namely the cognitive and emotional difficulties in response to climate change (reflected in worries about climate change, sleep disturbances, or nightmares about climate change) and the functional impairments associated with climate change anxiety (e.g., “My concerns about climate change interfere with my ability to do my work or schoolwork”, “My concern about climate change make it hard for me to have fun with my family or my friends”), with each of which, respectively, associated with potentially adaptive and maladaptive outcomes. Indeed, while the cognitive and emotional experience of climate change [e.g., worrying about climate change (Parmentier et al., 2024)] may help promote pro-environmental behaviors (e.g., reducing one's carbon footprint), the severity of the functional impairments has been viewed as the ultimate proxy for identifying when eco-anxiety becomes a significant threat to mental health (Heeren and Asmundson, 2023). Of clinical importance, research suggests that eco-anxiety in adolescents to be, in large proportion, characterized by functional impairments. For example, in a recent 10-country large-scale study in 10 countries (*n* = 10,000), more than 45% of young adults reported that eco-anxiety had severe, harmful effects on their daily functioning (Hickman et al., 2021). Yet, to our knowledge, the question of whether the functional impairments of eco-anxiety could lead to increased suicide risk in adolescents has never been investigated. This is unfortunate given the important predictive role of anxiety in adolescent suicide (e.g., Bentley et al., 2016) and the alarming rate of eco-anxiety in this group (including young adults) worldwide (Hickman et al., 2021; Tam et al., 2023).

Thus, in this study, our main objective was to clarify the relationship between eco-anxiety and the severity of suicide risk in adolescents. Following current research on eco-anxiety, we distinguished between the cognitive and emotional features of eco-anxiety from its functional impairment in daily life. Given the role of anxiety and depression in predicting suicide risk, we also examined the role of general anxiety and depression symptomatology in these relationships. Inspired by previous research, we predicted that the functional impairments associated with eco-anxiety might be a predictor of suicide risk in adolescents.

## 2 Methods

### 2.1 Participants

In this observational cross-sectional study with prospective recruitment, 87 patients from the child and adolescent psychiatric department of the Hospices Civils de Lyon, welcoming around 300 patients per year, were recruited from April 2023 to June 2023. We included patients from 12 to 16 years old due to contingent reasons (Supplementary material 1). All patients admitted to this short-term hospitalization department, mainly admitted after passing through a general emergency department, were screened for the study according to the (non-)inclusion criteria.

The criteria for non-inclusion were not speaking French (for adolescents), not being able to read or write, having an intellectual development disorder preventing potential comprehension of items or oral comprehension (e.g., severe dysphasia impeding the understanding of the explanations, or severe dyslexia impeding the reading of the items of the scales), refusing to participate (by the adolescent or by the holders of parental authority) or not being affiliated to a system of social security. Incomplete scales were dropped from the study. Participants were assessed for their demographic characteristics (age and sex—with a coding of 1 for men and 0 for women), and their primary psychiatric diagnosis (that led to their admission into the department). Primary psychiatric diagnoses are made on the basis of two concordant expert clinical interviews (AL and CG), according to the criteria of the Diagnostic and Statistical Manual of Mental Disorders, fifth edition (DSM-5). It should be noted that the gender declared by adolescents was collected for clinical reasons of diversity acceptance in their care environment; gender was not considered in the statistical analyzes. Treatments were not collected due to the complexity of psychiatric treatments, involving multiple medications, therapies, and interventions, and the wide variability in treatment options. Supplementary material 1 gives details on participants.

### 2.2 Number of participants

The number of participants required was calculated prior to the study on the basis of the analysis used as the main objective: a threshold of 85 patients was set, with a power calculation (using the pwr package) an effect size theoretically chosen at 0.15, based on an expected average effect (Green, 1991; Faul et al., 2007) justified by previous studies on eco-anxiety in relation to different psycho(patho)logical conditions (Clayton and Karazsia, 2020; Mouguiama-Daouda et al., 2022), with a significance level of 0.05 [effect size (*d* = 0.15), power (1-beta = 0.80), alpha (α = 0.05), and two-tailed test assumption]. Compared to the literature on suicide among hospitalized adolescent patients, the size of this group, which is particularly homogeneous, finely phenotyped and presents expected high values on the different psychopathology scales used (see below), is relatively large.

### 2.3 Procedure

During the first week of their hospitalization in the department, in the presence of a caregiver and with collection of written informed consent, participants completed three scales: the Columbia-Suicide Severity Rating Scale (C-SSRS) (Posner et al., 2011), the 13-item CAS scale (Clayton and Karazsia, 2020) and the Hospital Anxiety and Depression scale (HAD) (Zigmond and Snaith, 1983). We relied upon these measurement tools since they have, respectively, become among the most widely used ones worldwide, especially in youth and adolescent's. The total time required to administer the scales was about 30 min.

### 2.4 Ethics

This level-3 human research study was approved by the Sud Est II Personal Protection Committee on 30/03/2023 (ID-RCB No.: 2023-A00157-38) and conducted in accordance with the principles of the Declaration of Helsinki. The study was registered on an open-access clinical trials register (clinicaltrials.gov) before the inclusion of the first patient. According to the protocol requested by the ethics committee, the non-opposition of patients and their caregivers was collected with the collection of a written informed consent.

### 2.5 Measures

The 13-item CAS scale is a 13-item self-administered questionnaire validated in adults in 2020 (Clayton and Karazsia, 2020). It was translated and validated in French (Mouguiama-Daouda et al., 2022). To our knowledge, no scale on eco-anxiety has been specifically validated for children or adolescents. This scale is composed of two dimensions assessing: (i) cognitive and emotional difficulties in response to climate change, reflected through anxious ruminations, difficulty sleeping, concentration, crying and/or nightmares (eight items); ii) and functional impairment, with greater difficulty in socializing and/or concentrating in daily life, related to climate change (five items). Each item is rated on a 5-point ordinal Likert scale. There is no validated threshold for detecting clinically significant eco-anxiety using this scale. In Supplementary material 2, we also considered 2 other dimensions (integrating nine other items), which do not directly concern eco-anxiety, but were initially attached in addition to the 13-item CAS scale: the direct or indirect personal experience of climate change (“Climate Change Experience”), and the tendency to deploy adaptive behavioral responses to climate change (“Pro-environmental behavior”).

The C-SSRS is a main reference tool used in the international literature to assess the severity of suicide risk, suitable for adolescents from 12 years old (Posner et al., 2011). A linguistic validated translation has been conducted in French language (Fernandez et al., 2013; The Columbia Lighthouse Project, 2016). This validated semi-structured interview is a four-factor scale. The first factor measures the severity of ideation and consists of five items (5-point ordinal Likert scale). The second factor measures the intensity of ideation, composed of five items (5-point ordinal Likert scale). The third factor measures behavior and is rated on a nominal scale. The fourth factor measures lethality and current attempt (6-point ordinal Likert scale; if lethality is 0, potential lethality is scored on a 3-point ordinal scale). There is no validated threshold considering all the factors (total score that can vary from 0 to 40).

Finally, the HAD is a 14-item scale rated from 0 to 3, adapted to adolescents (Zigmond and Snaith, 1983), validated in French in adults only (Razavi et al., 1989; Bjelland et al., 2002). Seven questions are related to the anxiety dimension (HAD-A) and seven others to the depressive dimension (HAD-D). To detect anxious or depressive symptoms, the following interpretation can be proposed for each of the scores (HAD-A and HAD-D): absence of symptoms if 7 or less; doubtful symptomatology if 8–10; definite symptomatology if 11 or more. The internal reliability of the CAS was high in the study with a Cronbach's alpha of 0.89 for the global scale score (0.81 for the cognitive-emotional difficulties subscale and 0.82 for the functional impairments).

### 2.6 Statistical analysis

We provided the mean, median, standard deviation and minimum and maximum of the age, the number of males and females, of different gender described and of primary psychiatric diagnosis. We also provided the means, medians and standard deviations of the CAS total score, of the cognitive and emotional difficulties and functional impairment dimension scores, and of the C-SSRS total score. Specifically, for the CAS and its two dimensions, we provide the number and percentage above the median of the Likert scale score (more often than “sometimes”), and the number and percentage above the median of the subjects; for the HAD-A and the HAD-D, we give the number and percentage of subjects equal or superior to the cut-off of 11 and less than or equal to the cut-off of 7.

To respond more precisely to our hypothesis, we successively modeled the total score of the CAS (univariate model), then the dimensions of the CAS by considering the confounding factors (multivariate model). Thus, first, we seek to predict the total score of the C-SSRS (dependent variable) based on the total score of the CAS (independent explanatory variable), using a univariate linear regression (Supplementary material 2). Secondly, we seek to predict the C-SSRS total score based on its two dimensions separately (cognitive and emotional difficulties and functional impairment dimensions), anxiety (HAD-A) and depression (HAD-D), using a multivariate linear regression. We added to this multivariate model the age and sex. In parallel, we present the correlations (Spearman) between these variables in a heatmap plot.

We have standardized the beta coefficients to ensure consistency across different variable scales. Before proceeding with the analyses, we ensured the applicability conditions of the statistical tests were met (expected non-normality, floor and ceiling effects, presentation of Q-Q plots, skewness, and kurtosis) (Supplementary material 2).

All analyses were performed using R software (4.1.3). De-identified data and R script have been made publicly available via the Open Science Framework at https://osf.io/cnfrv/. Supplementary material 2, 3 give details on the models and methods.

## 3 Results

### 3.1 Description of the sample

Participants' characteristics are depicted in Table 1 and the CONSORT diagram is provided in Supplementary material 3. Among the 87 patients, 22 qualified for the DSM-5 diagnosis of mood disorder (major depressive episode = 18; bipolar disorder = 4), 16 for an isolated suicide attempt, 13 for the diagnosis of eating disorders (anorexia nervosa = 12; bulimia nervosa = 1), 11 for a diagnosis of neurodevelopmental disorder (Attention Deficit Hyperactivity Disorder = 7; Autism Spectrum Disorder = 4), nine for an emerging psychosis, four for a borderline disorder, three for a post-traumatic stress disorder, three for an Obsessive Compulsive Disorder, three for a behavior disorder, one for a gender dysphoria diagnosis, one for a generalized anxiety disorder and one for an anxious school refusal.

**Table 1:** Demographic characteristics (age, sex and declared gender), primary psychiatric diagnosis, scores on the Hospital Anxiety and Depression scale (HAD) for the anxiety dimension (HAD-A) and the depressive dimension (HAD-D), for the Climate Change Anxiety scale (CAS) and for the Colombia Suicide Severity Rating Scale (C-SSRS) (*N* = 87).

	**Variables**	
Demographic variables	Age	M = 13.79 (SD = 1.07); median = 14 [min. =12; max. = 16]
	Sex at birth	Male = 15; female = 72
	Gender	Woman = 64; Man = 16; nonbinary = 3; fluid = 2; transgender = 2
	Primary psychiatric diagnosis (15 different modalities)	Anorexia nervosa = 12; bulimia nervosa = 1; gender dysphoria = 1; MDE = 18; emerging psychosis = 9; anxious school refusal = 1; PTSD = 3; isolated suicide attempt = 16; generalized anxiety disorder = 1; bipolar disorder = 4; borderline disorder = 4; behavior disorder = 3; ADHD = 7; OCD = 3; ASD = 4
CAS	Total score	• M = 21.53 (SD = 8.49); Median = 21 • *Relative to the median of the Likert scale* (3): *N* = 3 (3.45%) • *Relative to the median of the subjects* (21): *N* = 38 (43.68%)
	Cognitive and emotional difficulties dimension	• M = 13.94 (SD = 5.65); Median = 13 • *Relative to the median of the Likert scale* (3): *N* = 7 (8.05%) • *Relative to the median of the subjects* (13): *N* = 41 (47.13%)
	Functional impairment dimension	• M = 7.57 (SD = 3.45); Median = 6 • *Relative to the median of the Likert scale* (3): *N* = 5 (5.75%) • *Relative to the median of the subjects* (6): N = 42 (48.27%)
C-SSRS	Total score	M = 18.48 (SD =12.20); Median = 24
HAD	Anxiety	• M = 12.76 (SD = 4.52); Median = 14 • *N* = 60 ≥ 11 (69.0%); *N* = 13 ≤ 7 (14.9%)
	Depression	• M = 9.71 (SD = 5.03); Median = 9 • *N* = 39 ≥ 11 (44.8%); *N* = 32 ≤ 7 (36.8%)

MDE, major depressive episode; PTSD, post-traumatic stress disorder; ADHD, attention deficit hyperactivity disorder; OCD, obsessive compulsive disorder; ASD, autism spectrum disorder; M, mean; SD, standard deviation; Min., minimum; Max., maximum.

### 3.2 Comparisons

Table 2 gives the results of the standardized model, considering the CAS total score (based on the univariate model), its two dimensions (cognitive and emotional difficulties and functional impairment dimensions), anxiety (HAD-A), depression (HAD-D), age, and sex (based on the multivariate model), to seek to predict the C-SSRS total score.

**Table 2:** Results of the standardized models predicting the Colombia Suicide Severity Rating Scale (C-SSRS) total score based on the Climate Anxiety Scale (CAS) total score (univariate model), its two dimensions separately (cognitive and emotional difficulties and functional impairment dimensions), anxiety (Hospital Anxiety and Depression – HAD-A), depression (Hospital Anxiety and Depression – HAD-D), age and sex (multivariate model).

		**β-coefficient**	***p*-value**
CAS	Total score	2.58	0.049^*^
	Cognitive and emotional difficulties	−0.35	0.81
	Functional impairment	0.45	0.76
HAD	Anxiety	3.16	0.02^*^
	Depression	6.15	< 0.001^***^
Demographic characteristics	Age	0.96	0.34
	Sex	−0.15	0.89

The *p*-value has been rounded from 0.04897.

*p*-value < 0.001: ^***^; *p*-value < 0.01: ^**^; *p*-value < 0.05: ^*^.

This model shows an adjusted *R*^2^ of 0.45 and an *F*_(6, 80)_ of 12.57. The cognitive and emotional difficulties dimension (β-coefficient = −0.35, *p* = 0.81) and the functional impairment dimension (β-coefficient = 0.45, *p* = 0.76) are not statistically associated with the severity of suicide risk. However, both anxiety (HAD-A, β-coefficient = 3.16, *p* = 0.02) and depression (HAD-D, β-coefficient = 6.15, *p* < 0.001^***^) show significant associations with suicide risk. Age and sex do not modify the significance of the results of this model [with a *p* = 0.34 (β-coefficient = 0.96) for age and a *p* = 0.89 (β-coefficient = −0.15) for sex].

Supplementary material 2 described this same standardized multivariate model with the total score of the CAS (and not its two sub-dimensions), which also does not show a significant relationship between suicide and eco-anxiety (*p* = 0.93). The diagnostic plots show no major deviations from normality or significant issues affecting the model's validity (Supplementary material 2).

**Figure 1:**
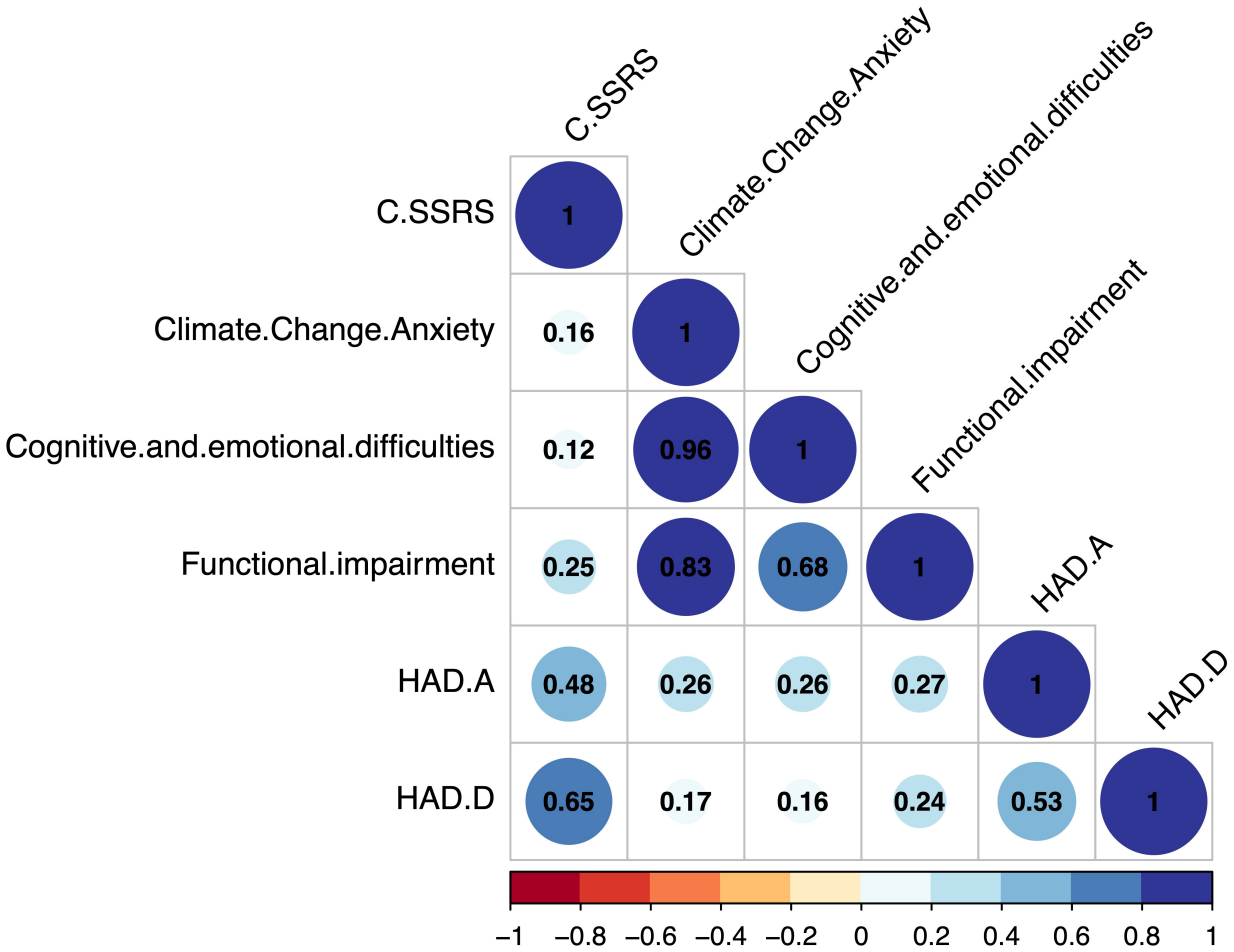
Heatmap of the correlations between the total scores of the C-SSRS and the Climate Anxiety Scale, the two dimensions of this scale (cognitive and emotional difficulties and functional impairment dimensions), and the total score of the HAD-A and HAD-D. CCA, Climate Change Anxiety scale (or CAS); C-SSRS, Colombia Suicide Severity Rating Scale; HAD-A/D, Hospital Anxiety and Depression scale (*N* = 87).

Finally, Figure 1 presents the heatmap of the correlations between the total score of the CAS, the two dimensions of the CAS separately, the total score of the C-SSRS and the anxiety (HAD-A) and depression (HAD-D).

## 4 Discussion

To the best of our knowledge, we have conducted the first study to examine the relationship between eco-anxiety and the severity of suicide risk in adolescents, as well as the first study of eco-anxiety conducted in a child and adolescent psychiatric unit.

Considered in isolation from other variables, a statistically significant association was found between the C-SSRS and the CAS. In this model, when eco-anxiety increases, the severity of suicide risk also increases. By being independent of depression and anxiety, this first univariate model is interesting because of the direct relationships between two societal and environmental hot topics: suicide and eco-anxiety. The interpretation and generalization of such a result should be carried out with great caution.

In the multivariate model, neither the cognitive and emotional difficulties dimension, nor the functional impairment dimension of eco-anxiety increase the severity of suicide risk. The absence of a significant relationship between sub-dimensions of the CAS warrants careful consideration to prevent overmedicalization of eco-anxiety. Indeed, interestingly in an expansive and varied literature, these results show for the first time that eco-anxiety may not be the priority of adolescents seen by adolescent psychiatrists in hospitalization. Such an observation would imply not overmedicalizing a dimension of life—certainly important for adolescents—which perhaps does not fall solely within the field of medicine. However, at the individual level, it remains ethically essential to remain vigilant, as certain cases could present heightened suicide risk even if population-level data do not reveal a clear connection (Nugent et al., 2019). Clinicians should continue to assess suicide risk with nuance, ensuring that potentially vulnerable individuals are not overlooked in clinical settings (Hughes et al., 2023).

Moreover, it is important to consider the potential mediating role of anxiety and depression in the relationship between eco-anxiety and suicide risk. In our model, the significant associations found between the HAD-A and HAD-D scores and suicide risk suggest that the psychological symptoms of anxiety and depression may partially explain this dynamic. This raises the possibility that eco-anxiety could indirectly influence suicide risk through its impact on these established mental health conditions. Further exploration of this mediation hypothesis, as well as more targeted studies, are necessary to clarify the pathways through which eco-anxiety interacts with broader psychological distress (Verplanken and Roy, 2013; Verplanken et al., 2020; Heeren and Asmundson, 2023).

In our sample, 3.45% of adolescents had eco-anxiety more often than “sometimes” on the CAS scale. These proportions were at 8.05 and 5.75% when the cognitive-emotional and functional impairment dimensions were, respectively, distinguished. Our results are significantly lower than proportions found in studies on adult populations in general (non-clinical) population: for instance, 11.64% of participants had eco-anxiety more often than “sometimes” in Heeren and collaborators, in the general adult population, with a proportion of 10.82 and 20.72%, respectively, for the cognitive-emotional difficulties and functional impairment dimensions (Heeren et al., 2022). Similarly, in Clayton and Karazsia (2020), 17–19% of participants in the general adult population had eco-anxiety more often than “sometimes” for the cognitive and emotional difficulty dimension, and 26–27% for the functional impairment dimension. These prevalence could be different from those of our cohort for at least two main reasons: either because they come from the general population, while our cohort is clinical; or because they come from an adult population, while our cohort concerns adolescents. Finally, in line with other studies (Mouguiama-Daouda et al., 2022), suicide risk is well-associated with anxiety and depression.

More generally, too few empirical articles on the eco-anxiety of young people are conducted, especially under the age of 18. A selective analysis targeted on eco-anxiety, conducted empirically within two systematic reviews on the impact of climate change on young people (Léger-Goodes et al., 2022; Martin et al., 2022), found 44 articles on eco-anxiety in children and/or adolescents and/or young adults under the age of 25 years (listed in Supplementary material 4). Eco-anxiety is mainly described as “important” in young adults, as described by Hickman et al. (2021) in 10,000 children and young people in ten countries, with 50% reporting negative emotions and 45% admitting that their feelings about climate change “negatively affected their daily life and functioning”. In the same way, a survey carried out in Australia among 600 children aged 10–14 revealed that 44% of children were worried about the future impacts of climate change (Tucci et al., 2007).

This study has some limitations. First, in our cohort, we find a lower prevalence of eco-anxiety than in these other international studies of young people. There are at least three explanations for this difference. It could be related to our sample with an imbalance and females and/or a relatively young mean age (13.79 years), which could explain lower levels of eco-anxiety and age independence than in other studies; second, comparing eco-anxiety data across studies could be difficult because of heterogeneity in the measurements tools used so far (for a discussion, see Martin et al., 2022); third, these differences in prevalence may be related to the fact that our study focuses on patients hospitalized in a child psychiatric unit, while other did not. This is an important result in itself since no study, to our knowledge, has been based on a group of hospitalized adolescents—despite the relative psycho(patho)logical vulnerability of this population. Other methodological limitations of this study should be noted, in particular the lack of an eco-anxiety scale specifically validated for children and adolescents, the non-collection of some sociodemographic data (e.g., urban or rural residence or socioeconomic level of parents), that may modulate our results.

## 5 Conclusion

Suicide is a major public health concern, and anxiety is a significant risk factor for suicide in adolescents; eco-anxiety, stemming from climate change concerns, is a growing source of distress in this specific population and can lead to harmful mental health consequences. Considering suicide in adolescents within the scientific domain of eco-anxiety opens avenues of research at the crossroads of environmental and mental health sciences. This study aims to explore, for the first time to our knowledge, the relationship between eco-anxiety and suicide risk in adolescents, with a focus on the impact of functional impairments. These findings suggest that eco-anxiety might not be the main concern for adolescents in the care of adolescent psychiatrists. However, we strongly emphasize the need to continue the evaluation of such associations between psychiatric disorders and eco-anxiety, advocating for a minimal ethical and prudent approach in mental health care for this population.

## Data Availability

The datasets presented in this study can be found in online repositories. The names of the repository/repositories and accession number(s) can be found below: https://osf.io/cnfrv/.
