# Supplementary material for: Exploring the relationship between eco-anxiety and suicide risk in adolescents with mental health disorders: insights from a cross-sectional observational study
Source: Front Psychol. 2025 Jan 7;15:1408835. doi: 10.3389/fpsyg.2024.1408835 (PMC11747365; doi:10.3389/fpsyg.2024.1408835)
Supplement: Supplementary file 1 [file Data_Sheet_1.pdf]

Supplementary Material:

Exploring the relationship between eco-anxiety and suicide risk in adolescents with mental health disorders: Insights from a cross-sectional observational study

1 Supplementary Material 1

Definition of adolescence

According to the International Council for Harmonisation of the European Medicines Agency (EMA-ICH), we define adolescence as the “12-18 years” period, in which we have only studied adolescents from 12 to 16 years old. The department only accepts patients between the ages of 12 and 16, and it was not possible to have young people between the ages of 16 and 18, despite the definite interest in including this population. Furthermore, the age of 12 corresponds to the threshold of age proposed in the validation of the C-SSRS and the CAS scales.

Calculation of the number of subjects

A threshold of 85 patients was set, with a power calculation using the pwr package (i.e., basic functions for power analysis), considering u = 5 (numerator degrees of freedom), f2 = effect size (theoretically chosen at 0.15 based on based on an expected average effect (Faul et al., 2007; Green, 1991), justified by previous studies on eco-anxiety in relation to different psycho(patho)logical conditions (Clayton & Karazsia, 2020; Mouguiama-Daouda et al., 2022), sig.level = 0.05 (level of significance), power = 0.80. Given that this exploratory study, including strong ethical issues, did not hypothesize the existence of a relationship between suicide, eco-anxiety and primary diagnosis, the calculation of the number of required subjects has not integrated the latter (which would have required the integration of approximately 140 to 280 additional subjects, raising feasibility issues).

Regulatory aspects

The non-objection was collected orally from the participant and the holders of parental authority during the presentation of the study, and traced in writing in the medical file, after the form was given to the participant and the holders of the parental authority. Parents who did not speak French were not a criterion for non-inclusion, because the scales (validated in French) were only self-questionnaires directly concerning the children, and the study could be explained to the parents in another language than French, with an approved translator (as is usually practiced in the department in this case), in order to collect their non-objection. There were no parents who did not speak French in the recruitment.

Data were entered into a spreadsheet, anonymously, for blinded statistical processing.

2 Supplementary Material 2

1. Details of scales and subscales

- The C-SSRS-screen includes the 5 items of the C-SSRS on the severity of suicidal ideation, and a 6th item on the history of suicidal behavior with a threshold greater than or equal to 3 (Bjureberg et al., 2021). The optimal threshold of the C-SSRS-screen for the severity of suicide risk is greater than or equal to 3 points (sensitivity of 53.9%; specificity of 75.6%; Odd-Ratio (OR) of 3.6 with multiplication four times the risk of dying by suicide at 1 week and 1 month and doubling the risk of dying at one year) (Bjureberg et al., 2021).

- The table below presents the mean, median, and standard deviation for the two dimensions of the CAS: cognitive and emotional difficulties, and functional impairment.

Dimension	Mean	Median	Standard Deviation
**Cognitive and Emotional Difficulties**	**13.95**	**13**	**5.65**
**Functional Impairment**	**7.57**	**6**	**3.44**

Three adolescents (3.5%) had a mean CAS score above the median of the scale (i.e., at 21). Six adolescents (6.9%) had a mean score above the median of the scale for the cognitive and emotional difficulties dimension. Four adolescents (4.6%) had a mean score above the median of the scale for the functional impairment dimension. Fifty-nine adolescents had a C-SSRS screen score greater than 3 (67.81%).

- Individuals with a mean score below the median of the scale are considered to have low eco-anxiety and those with a mean score above the median of the scale are considered to have high eco-anxiety (Heeren et al., 2021). A score for each subscale can also be calculated. Two other subscales are also added to the scale, assessing climate change

experience (with a Cronbach's alpha of 0.78) and pro-environmental behavior (with a Cronbach's alpha of 0.69), but are not part of the main comparison analysis.

- We analyzed the means, medians and standard deviations of the four dimensions of the C-SSRS. We also calculated the number of participants whose C-SSRS screen threshold was greater than 3 (**Table below**).

**Dimension C-SSRS**	**Mean**	**Median**	**Standard Deviation**
Suicidal ideation	3.82	5	2.47
Severity of ideation	12.51	15	8.39
Suicidal behavior	2.67	3	1.94
Lethality	0.02	0	0.15

2. Details on the main models

Using the "scale" function allowing to standardize variables, the standardized univariate linear model was written as follows:

$$\boldsymbol{lm}(\boldsymbol{formula} = \boldsymbol{Ecoanxiety}\$\boldsymbol{C.SSRS} \sim \boldsymbol{Ecoanxiety}\$\boldsymbol{Climate.Change.Anxiety})$$

with lm for `linear model`, and the `C.SSRS` as the depend variable with the `Climate.Change.Anxiety` as the independ variable.

The multiple linear model was written as follows:

$$\begin{aligned} \boldsymbol{lm}(\mathbf{Ecoanxiety\$C.SSRS} \sim\ & \mathbf{Ecoanxiety\$Cognitive.and.emotional.difficulties} \\ & + \mathbf{Ecoanxiety\$Functional.impairment} + \mathbf{Ecoanxiety\$HAD.A} \\ & + \mathbf{Ecoanxiety\$HAD.D} + \mathbf{Ecoanxiety\$Age} + \mathbf{Ecoanxiety\$Sex}) \end{aligned}$$

with lm for `linear model`, and the `C.SSRS` as the depend variable with, as the independ variables: `Cognitive.and.emotional.difficulties`, `Functional.impairment`, HAD.A, HAD.D, Age & Sex.

3. Complementary models

Model A: Univariate CAS-CSSRS model – In the standardized univariate model, a statistically significant relationship was found between the C-SSRS and the CAS ($F(1,85)= 3.99$; $p = 0.049$; β-coefficient = 2.58 [0.01 – 5.14); adjusted-$R^2 = 0.03$]).

Model B: Multivariate CAS-HAD-CSSRS model – We then conducted a multivariate model to explore the C-SSRS, using the total CAS score (without its sub-dimensions), and HAD-A and HAD-D as predictors. Thus, this model examines the C-SSRS in relation to the CAS, HAD-A, and HAD-D. In this model, the total CAS score did not show a significant effect ($p = 0.92$). However, both HAD-A and HAD-D were significant predictors, suggesting their contribution to the model's overall significance ($F = 0.81$, $p = 0.01$ for HAD-A, and $F = 1.26$, $p < 0.01$ for HAD-D).

Model C: Ordinal CAS-CSSRS model – Thus, we compared the quantitative C-SSRS scores with the quantitative CAS scores. We also studied the association between the C-SSRS screen (threshold at 3) and the CAS (threshold established according to the mean score below the median of the scale). To meet this secondary objective, on the basis of a C-SSRS screen threshold greater than or equal to 3, we divided the patients according to the severity of suicide risk (1 = risk; 0 = no risk) and according to the intensity of eco-anxiety (1 = high eco-anxiety; 0 = low eco-anxiety). In order to study the association of two ordinal independent variables (C-SSRS and CAS) to 2 groups (1 and 0), we uses a $\chi 2$ test. This model was also corrected for HAD scores, age, sex and main psychiatric diagnosis. We checked the conditions for applying such a test: independence of errors, linearity in the logit for continuous variables, absence of multicollinearity and absence of strongly influential outliers. We used the lme4 (R) package and the lme function. We also used a logistic regression to compare the C-SSRS-screen and the quantitative CAS scores, a model which was also corrected for the HAD scores, age, gender and primary psychiatric diagnosis.

Model D: Subscales and covariates model – Then, we modelled the associations between the C-SSRS and the 13-item CAS by considering the two additional subscales of the 22-item CAS (climate change experience and pro-environmental behavior) (multiple linear regression). We used a multiple regression to study the linear relationship between the CAS and these two subscales. More precisely, we conducted a complementary model including all predictors to examine the relationship between the C-SSRS and: CAS, HAD-A, HAD-D, age, sex. The significant relationship between the CAS and the C-SSRS remains when controlling for age ($p = 0.04$; β-coefficient = 0.12 [0.006–0.242]) and sex ($p = 0.03$; β-coefficient = 0.13 [0.008–0.243]). Similarly, significant relationship persists when controlling for the HAD ($F = 3.14$, $p = 0.024$), specifically for the HAD-A ($p = 0.047$; β-coefficient = 0.49 [0.006–0.975]), but not for the HAD-D ($p = 0.537$; β-coefficient = 0.15 [-0.335–0.638]).

Model E: Threshold-based C-SSRS screen-CAS model – The comparison between the C-SSRS-screen and the CAS (according to the established threshold) does not appear significant ($\chi 2 = 0.300$; $p = 0.584$). No positive relationship was found when using a threshold for the severity of suicide risk with the C-SSRS screen and a threshold for the CAS (using the median score of the scale as a threshold – see (Clayton & Karazsia, 2020; Heeren et al., 2021)). However, this lack of difference could be explained by the fact that few adolescents presented eco-anxiety in the high norm of the scale within our cohort (M = 21.53; Median = 21; ET = 8.49). Indeed, 3.45% of adolescents had

higher eco-anxiety than the median; this proportion was 8.05% and 5.75% when the dimensions evaluating cognitive-emotional difficulties and functional impairment are distinguished respectively. These first results open new avenues considering the study of dimensions and subscales through the prism of a validated threshold of the CAS.

Model F: Logistic regression CAS-CSSRS screen model – The logistic regression shows a significant difference between the C-SSRS-screen (dependent variable) and the quantitative CAS scores (independent variable) ($p = 0.032$; β-coefficient for the CAS = 0.013 [0.001 – 0.024]), which however disappears when corrected by the HAD (with a very significant relationship between the depressive dimension and the C-SSRS-screen, $p < 0.001$; β-coefficient for the HAD-D = 0.038 [0.019 – 0.057]).

Model G: CAS-HAD association model – We also confirm that CAS is strongly associated with the HAD-D ($F = 5.277$; $p = 0.024$; β-coefficient = 0.408 [0.055 – 0.761]) as well as HAD-A ($F = 9.49$; $p = 0.003$; β -coefficient = 0.596 [0.211 – 0.980]). This relationship between eco-anxiety and depression is consistent with the international literature (Mouguiama-Daouda et al., 2022). The relationship between eco-anxiety and anxiety is found in a variable way in the literature: authors such as Clayton and Karazsia (2020) certainly found a correlation ($r = 0.60$ and $r = 0.54$ for the dimension of cognitive and emotional difficulties; $r = 0.56$ and $r = 0.47$ for the functional impairment dimension), but the measurement of depression and anxiety was not dissociated. On the contrary, Mouguiama-Daouda and collaborators (2022) did not find any correlation using only a measure relating to generalized anxiety (using the GAD-7 (Generalized Anxiety Disorder) (Spitzer et al., 2006) ($\rho = 0.02$ [–0.09 to 0.12])). This relationship between eco-anxiety and anxiety-depression is particularly important to consider in future studies.

Model H: CAS subscales-CSSRS model – Finally, we find significant associations between the C-SSRS and the 13-item CAS when considering separately the two other subscales of the 22-item CAS ($F = 22.48$; $p < 0.001$ β-coefficient for the "Climate change experience" subscale = 1.27 = [0.779 – 1.722] and β-coefficient for the "Pro-environmental behavior" subscale = 0.469 [0.148 – 0.789]).

The results highlight significant relationships between the CAS and the C-SSRS, particularly when considering specific subscales and controlling for variables such as age, sex, and the two HAD

dimensions. However, threshold-based comparisons yielded limited findings, likely due to the low proportion of adolescents presenting high levels of eco-anxiety in this cohort.

4. Description of regression applicability conditions and information on non-normality

The residuals / fitted plot (**Figure 1**) shows the relationship between residuals and fitted values, allowing to detect any non-linearity or patterns in the residuals. In our case, the residuals appear to be randomly distributed around the zero line, which suggests no significant violations of the linearity assumption. Any notable pattern or curvature in this plot would have indicated potential misspecifications in the model or non-linearity, but this was not observed here.

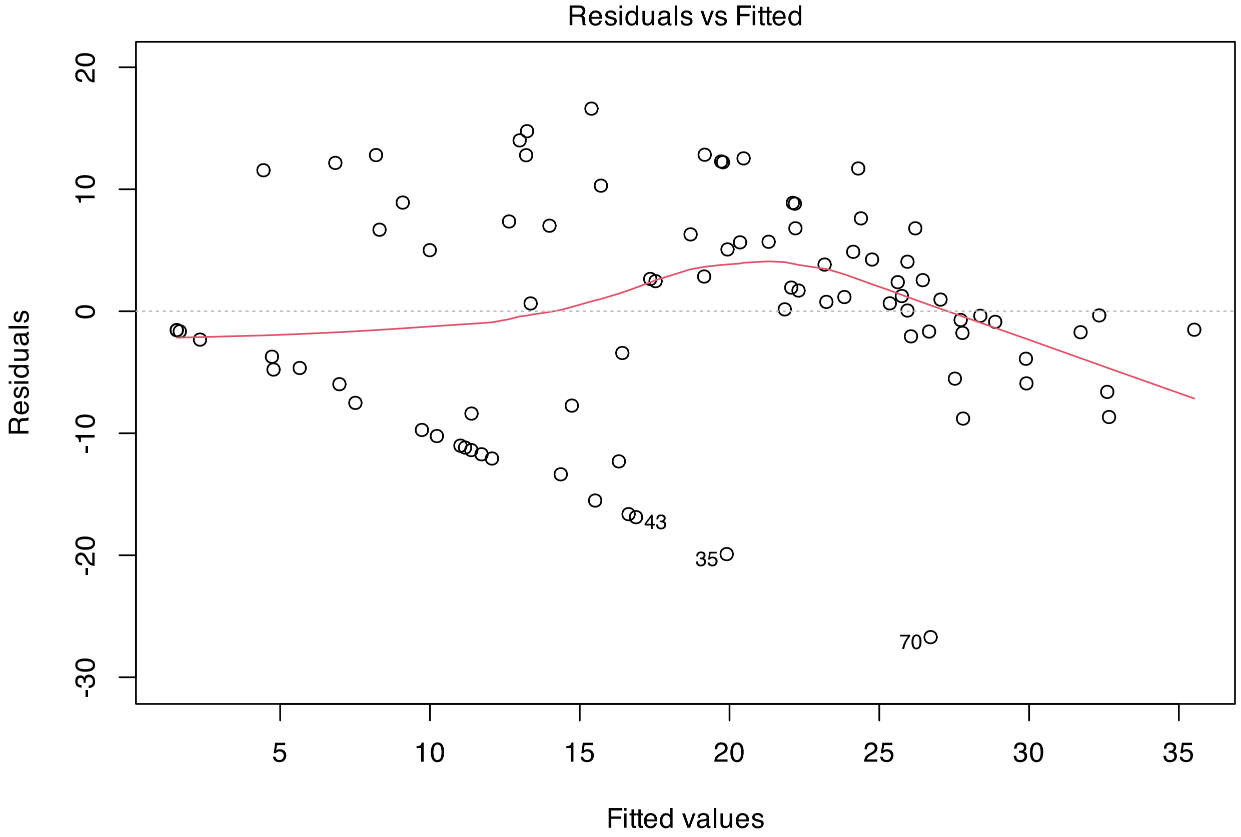

The Q-Q plot (**Figure 2**) compares the standardized residuals to a theoretical normal distribution. In this plot, most points align well with the diagonal reference line, confirming that the residuals are approximately normally distributed. Deviations from this line, particularly at the extremes,

could suggest problems with non-normality, but here, only minimal deviations are present, indicating that the assumption of normality holds.

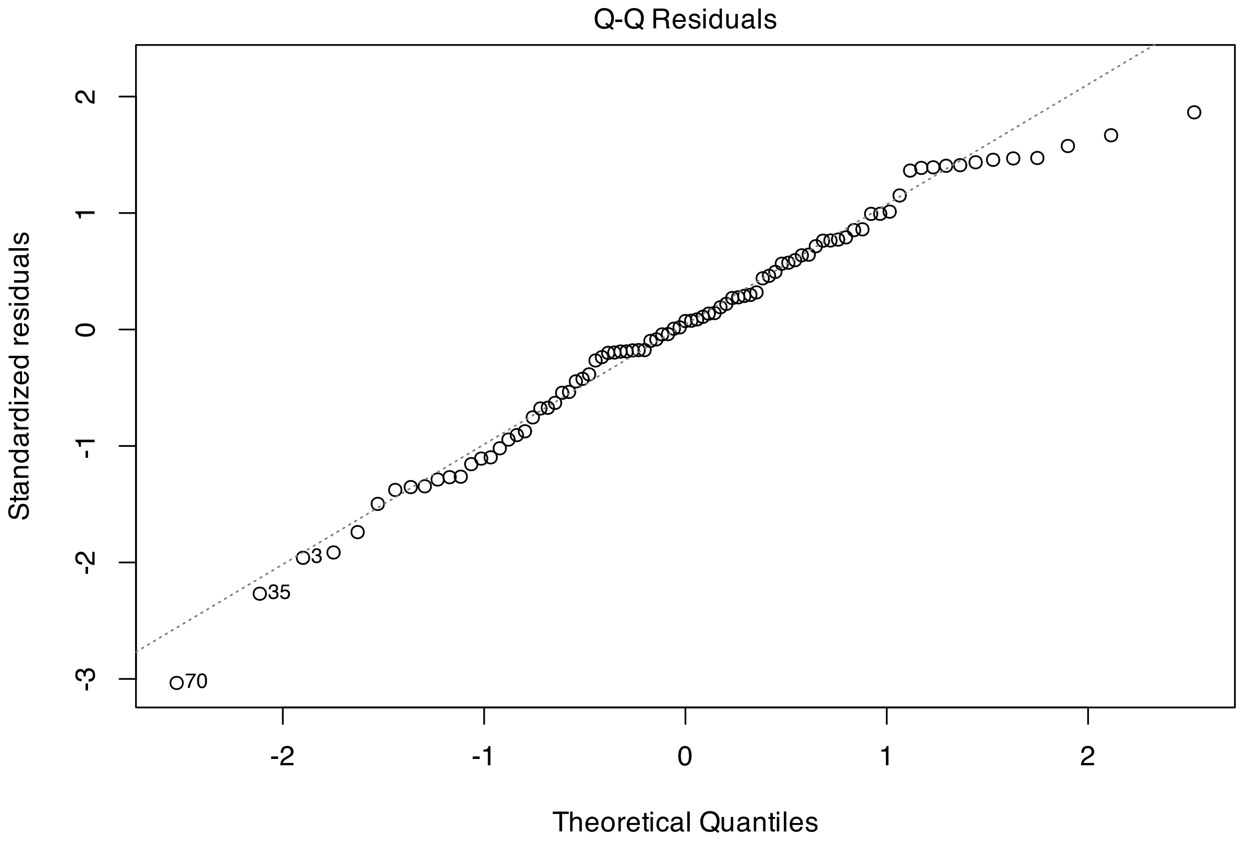

Finally, the scale-location plot (**Figure 3**) examines the homoscedasticity assumption by plotting the standardized residuals against the fitted values. The points in this plot are evenly spread, with no clear pattern, supporting the conclusion that the variance of residuals is roughly constant across

all levels of the fitted values. This confirms the assumption of homoscedasticity, as any visible trend (such as a funnel shape) would have indicated heteroscedasticity.

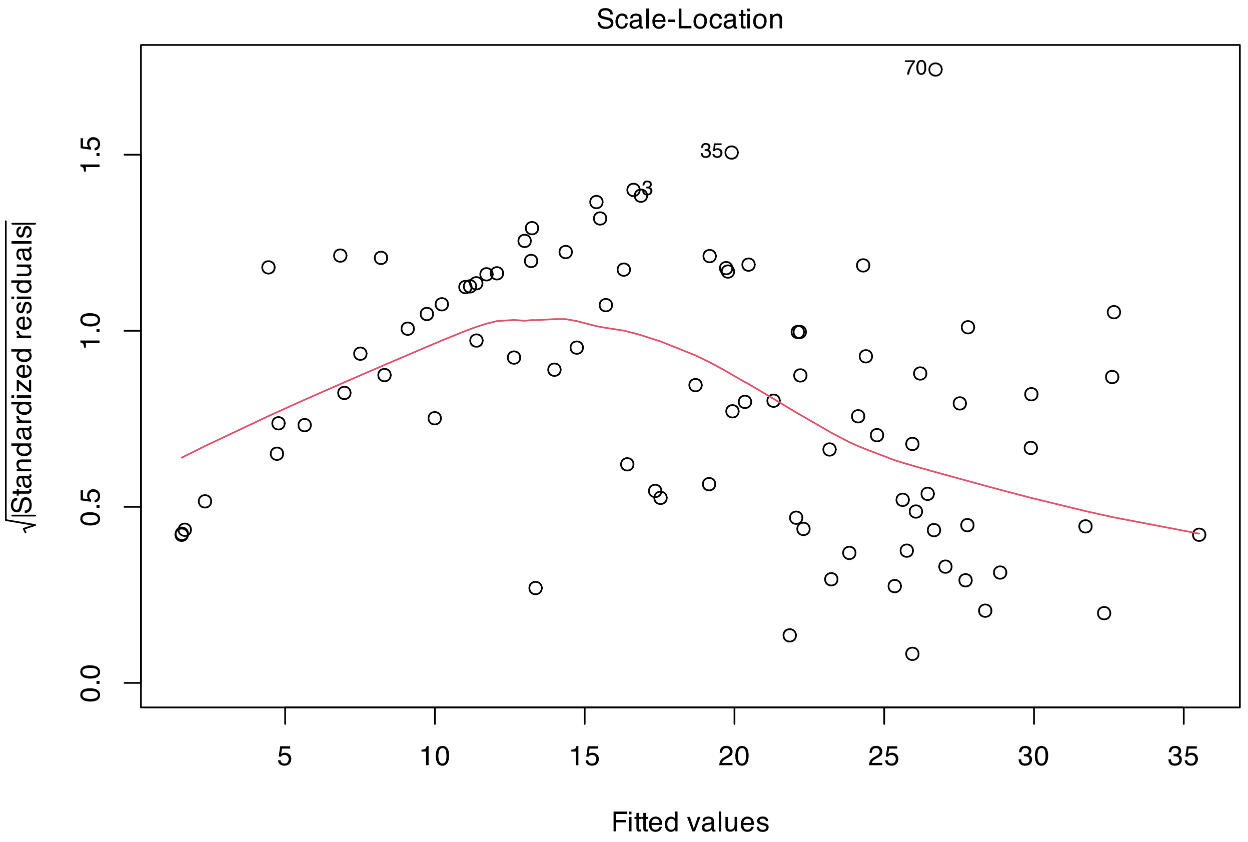

These diagnostic plots provide confidence that the key assumptions of linear regression – linearity, normality, and homoscedasticity – are sufficiently met in our model.

3 Supplementary Material 3

CONSORT flow diagram for cross-sectional study on eco-anxiety and suicide.

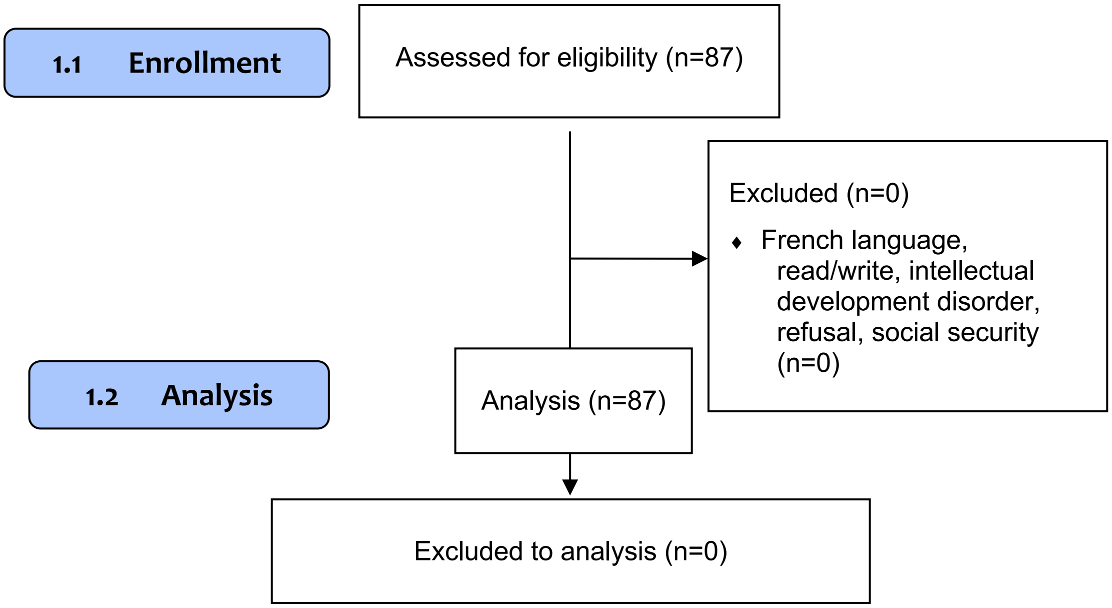

4 Supplementary Material 4

A targeted analysis focusing on eco-anxiety, conducted empirically as part of two systematic reviews on the impact of climate change on young people (Léger-Goodes et al., 2022; Martin et al., 2022), found 44 articles on eco-anxiety in children, adolescents, and/or young adults under the age of 25:

Baker et al., 2020 ; Bangsund, 2018 ; Boggs et al., 2016 ; Burke et al., 2018 ; Chalupka et al., 2020 ; Clayton et al., 2017 ; Clayton, 2020 ; Clemens et al., 2020 ; Cunsolo et al., 2020 ; Fritze et al., 2008 ; Galway et al., 2019 ; Gifford & Gifford, 2016 ; Harker-Schuch et al., 2021 ; Hickman et al., 2021 ; Huang & Yore, 2005 ; Kowalczewski & Klein, 2018 ; Kuang & Root, 2019 ; Li & Monroe, 2019 ; MacKay et al., 2020 ; McMichael, 2014 ; Nagel, 2005 ; Ojala, 2012b, 2012a, 2013, 2016 ; Ojala & Bengtsson, 2018 ; Palinkas & Wong, 2020 ; Pinsky et al., 2020 ; Pinto & Grove-White, 2020 ; Plautz, 2020 ; Ratinen & Uusiautti, 2020 ; Sanson et al., 2018, 2019 ; Sobel, 2007 ; F. Stanley & Farrant, 2015 ; Stevenson & Peterson, 2015 ; Strife, 2012 ; Strohmeier et al., 2017 ; Taylor & Murray, 2020 ; The Lancet Child & Adolescent Health, 2021 ; Tucci et al., 2007 ; UNICEF, 2013 ; Wu et al., 2020 ; Zummo et al., 2020.
